# Epigenetics in the wild

**DOI:** 10.7554/eLife.07808

**Published:** 2015-05-05

**Authors:** Adam J Bewick, Robert J Schmitz

**Affiliations:** Department of Genetics, University of Georgia, Athens, United States; Department of Genetics, University of Georgia, Athens, United Statesschmitz@uga.edu

**Keywords:** epigenetics, population genetics, local adaptation, DNA methylation, *Arabidopsis*

## Abstract

Studies of wild populations of the model plant *Arabidopsis thaliana* have started to reveal how patterns of DNA methylation change in response to the local environment.

**Related research article** Dubin M, Zhang P, Meng D, Remigereau MS, Osborne E, Casale FP, Drewe P, Kahles A, Jean G, Vilhjálmsson B, Jagoda J, Irez S, Voronin V, Song Q, Long Q, Rätsch G, Stegle O, Clark R, Nordborg M. 2015. DNA methylation in *Arabidopsis* has a genetic basis and shows evidence of local adaptation. *eLife* **4**:e05255. doi: 10.7554/eLife.05255

**Image** Much of the variation in CHH DNA methylation can be linked to alleles in one region of the genome (shaded vertical bar)

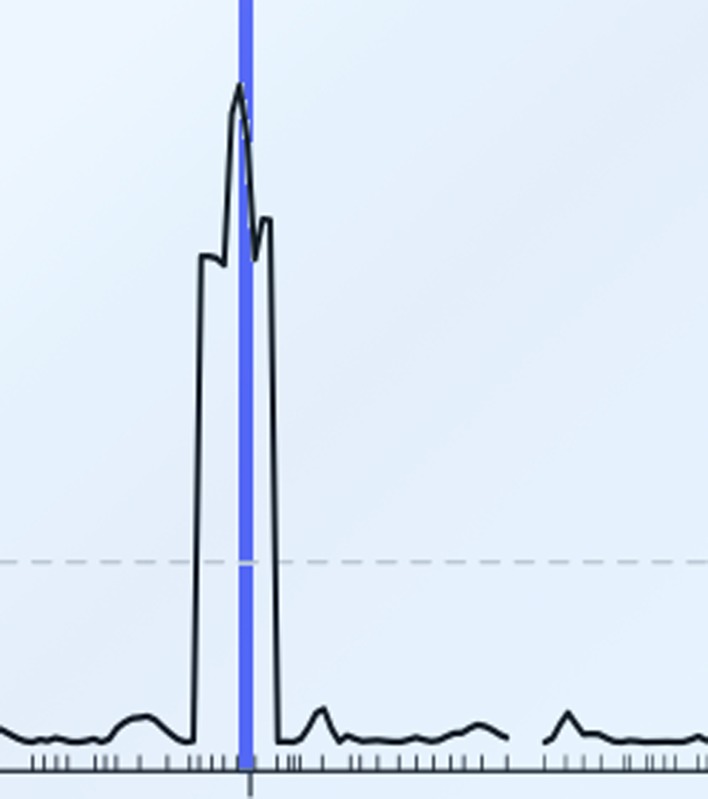

Individuals of the same species often carry the same genes but with slight differences. Each version of a gene is called an allele, and individuals with certain alleles can display certain traits or characteristics that will give them, within their local environment, a fitness advantage over individuals with different alleles. How genes underlie this ‘local adaptation’ and how natural selection shapes this process have been actively researched since the middle of the last century (Orr, 2005). In recent years, however, it has emerged that there are heritable traits that are not the direct result of differences in DNA sequences. These epigenetic variations can also provide the raw materials for natural selection to occur (Eitchen et al., 2014).

DNA molecules can be very long; as a result they are wrapped around proteins called histones so that they can be easily packed inside a cell's nucleus. Both DNA and the histone proteins can be chemically modified, and alleles with the same DNA sequence but different patterns of chemical modifications (called ‘epialleles’) can be passed between generations and contribute to complex traits or characteristics (Cortijo et al., 2014). Now in *eLife*, Magnus Nordborg and co-workers—from Austria, the US, the UK and Germany—have explored variations in DNA methylation among wild populations of a plant called *Arabidopsis thaliana* in Sweden to see how epigenetic variation is influenced by the local environment (Dubin et al., 2015).

Methylation is a chemical modification to DNA that inhibits the proliferation of selfish DNA elements (such as transposable elements) and helps regulate gene expression. Several proteins and enzymes work together in pathways to establish and maintain DNA methylation at sites with one of the following DNA sequences: CG, CHG (where H can be an A, T or C base) or CHH (Law and Jacobsen, 2010).

One pathway is responsible for ‘gene body methylation’, which involves the methylation of DNA within a large subset of genes, but only at CG sites. However, transposable elements can be methylated by multiple pathways: one pathway important to this study involves the enzyme CMT2, which methylates long or ‘deep’ transposable elements at CHG and CHH sites (Zemach et al., 2013; Stroud et al., 2014). In this case, ‘deep’ refers to transposable elements that are within tightly packed (or heterochromatic) regions of the genome.

Patterns of DNA methylation at regions within both genes and transposable elements vary extensively within and among natural *Arabidopsis* populations (Schmitz et al., 2013). However, the potential effects of this epigenetic variation on fitness and local adaptation remain unclear. Nordborg and co-workers—who include Manu Dubin, Pei Zhang, Dazhe Meng and Marie-Stanislas Remigereau as joint first authors—found that DNA methylation at CHH sites in transposable elements increases with temperature (Dubin et al., 2015, Figure 1A). Using CHH methylation as a trait, Nordborg and co-workers then conducted a genome-wide search and revealed that, in general, a lot of the variation in this trait could be explained by which allele the plant carried at a specific site called *CMT2a* (Figure 1B). This site is near a gene that encodes an enzyme called CMT2, which is known to methylate CHG and CHH sites in long transposable elements (Zemach et al., 2013; Stroud et al., 2014). All the plants examined carried one of two possible alleles (that differed by a single DNA base). Furthermore, plants with the less common of the two alleles—called the ‘non-reference allele’—typically had more CHH methylation than plants with the more common reference allele. Additional searches revealed another similar site nearby, called *CMT2b*. In this case plants with the rarer non-reference allele had less CHH methylation on average.

**Figure 1.**
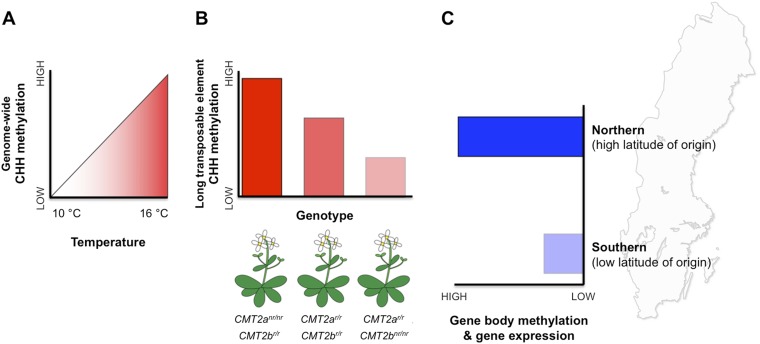
DNA methylation responds to temperature changes. (**A**) Methylation at CHH sites across the whole genome is higher in plants that are grown at higher temperatures. (**B**) Individual plants with different genotypes (that is, in plants with different combinations of alleles) for the gene that encodes the CMT2 enzyme show different levels of CHH methylation in long transposable elements. For example plants with non-reference (nr) alleles at *CMT2a* and reference (r) alleles at *CMT2b* display a high rate of CHH methylation. (**C**) Plants originating from northern regions of Sweden (high latitude of origin) have higher levels of gene-body methylation and overall gene expression than plants originally from southern Sweden.

The two pairs of alleles were found in populations of *Arabidopsis* from both southern and northern Sweden, but the non-reference alleles were more common in southern regions. This may indicate that there is gene flow between populations, or that natural selection is still ‘in action’ and continues to select for one allele over the other but has not yet ‘fixed’ the alleles between populations. Together with other recent results (Shen et al., 2014), these latest findings indicate that the temperature-dependent CHH methylation is a flexible trait, and that certain alleles that encode the CMT2 enzyme may make plant genomes more responsive to environmental changes.

In addition to CHH methylation, Nordborg and co-workers also observed a correlation between gene body methylation and the latitude of origin (Figure 1C). Specifically, populations from northern regions had higher levels of gene body methylation. Genes that are more heavily methylated in the northern regions are expressed at higher levels compared to their less methylated counterparts in the south.

This work is a first step on the way to a full understanding of how environment and genetic makeup contribute to the variation in DNA methylation observed in wild populations. The work also suggests that genetic variation at enzymes involved in DNA methylation may provide some populations with an advantage to changing environmental conditions or seasons.

Future experiments, including moving wild plants between different populations and then assessing their fitness, would shed more light on DNA methylation and its role in local adaptation. Likewise, comparisons between individuals of different species could unveil other types of naturally occurring diversity and provide a wealth of genetic or genomic resources to help us better understand DNA methylation in the light of evolutionary biology. Furthermore, studies within a species could help determine how much variation in physical traits is controlled by DNA methylation variation as opposed to genetic variation. In this scenario each genome-wide difference in DNA methylation is used as a marker and tested for an association with the trait under study. However, differences in DNA methylation markers between different populations due to demographic factors would confound these studies, making it difficult to determine the underlying epigenetic variation that contributes to the traits.

Research into DNA methylation (and epigenetics in general) has only recently begun in the natural sciences. Understanding the relationship between an organism's fitness and its epigenetics, traits and environment represents a challenging, but fruitful, area of future research.
